# Assessing the viability of heart rate variability as an objective and comprehensive indicator of chronic non-specific neck pain

**DOI:** 10.1371/journal.pone.0326357

**Published:** 2025-07-02

**Authors:** Chao Zhang, Hai-Peng Xu, Hui-Fang Zheng, Xiao-Lian Wu, Hong-Gen Du, Xin Jin

**Affiliations:** 1 Department of Rehabilitation, The First Affiliated Hospital of Zhejiang University of Traditional Chinese Medicine, Hangzhou, Zhejiang, China; 2 Department of Tuina, The First Affiliated Hospital of Zhejiang University of Traditional Chinese Medicine, Hangzhou, Zhejiang, China; 3 Department of Health Management Center, The First Affiliated Hospital of Zhejiang University of Traditional Chinese Medicine, Hangzhou, Zhejiang, China; Kasetsart University Faculty of Veterinary Medicine Kamphaengsaen Campus, THAILAND

## Abstract

**Background:**

This study investigates heart rate variability (HRV) in chronic nonspecific neck pain (CNNP) patients, revealing reduced HRV linked to pain intensity, anxiety, and cervical instability, and proposes HRV as an objective biomarker for comprehensive CNNP assessment.

**Method:**

According to the inclusion and exclusion criteria, 80 patients were included in the experimental group. In addition, 80 healthy individuals matched for age, sex, and other basic information were selected as the control group. The clinical outcome of patients was assessed using the visual analog scale and the self-rating anxiety scale. Subsequently, imaging studies were performed to assess cervical stability (atlantoaxial deviation, axis rotation), osteophytes, physiologic curvature, and disc herniation in patients. The frequency domain (low frequency, high frequency, and their ratio) and time domain (standard deviation of all normal-to-normal QRS intervals) HRV indexes were obtained from all subjects using the heart rate meters.

**Results:**

All HRV indexes were significantly lower in patients compared to controls, and pain and anxiety further reduced HRV indexes in patients. Pain intensity, anxiety, and disc herniation were significantly correlated with all the HRV indexes. Mild instability was not correlated with the HRV, but instability reaching a certain level (atlantoaxial deviation >0.1 mm or rotation of the axis >2 mm) was significantly correlated with the HRV indexes.

**Discussion:**

Chronic nonspecific neck pain associated with autonomic nervous system dysfunction, and heart rate variability provides an objective and comprehensive assessment of chronic nonspecific neck pain.

## Introduction

Chronic nonspecific neck pain (CNNP) is defined as pain in the neck and/or shoulder girdle region for a duration of more than 12 weeks in the absence of a clear etiological cause [1]. The prevalence of CNNP is high, with a global prevalence rate of 3551.1 per 100,000 people [2]. CNNP is also characterized by a high relapse rate and costly treatment, resulting in physical and mental disability and a decline in patient health status [3]; the condition is a public health issue and imposes a high global burden [4].

Recent studies have shown that CNNP was associated with an elevated incidence of cardiovascular and cerebrovascular adverse events, with acute coronary syndromes (ACS) occurring 1.13 times more frequently in CNNP patients than in non-CNNP patients, and posterior ischemic strokes occurring 1.46 times more frequently in CNNP patients than in non-CNNP patients [5,6]. It had been shown that the occurrence of cardiovascular events was closely linked to the regulation of the autonomic nervous system (ANS). From the above references, it appeared that CNNP may increase the incidence of cardiovascular disease. Therefore, CNNP might be associated with autonomic nervous system (ANS) dysfunction. However, few studies had investigated the ANS function in CNNP patients, and even fewer have explored the factors associated with ANS dysfunction in CNNP patients.

Heart rate variability (HRV) refers to the variation of R-R intervals in the sinus beats of the heart, which is jointly regulated by the sympathetic and parasympathetic nerves, and may reflect the functional status of the ANS [7]. As a non-invasive, rapid, and quantitative indirect measure of autonomic function, HRV is widely used for risk assessment in patients with cardiovascular disease. Previous studies [8] have confirmed that, within a certain range, higher HRV is associated with stable ANS function. In contrast, a decrease in HRV parameters reflects abnormalities in the ANS and is closely related to adverse events such as myocardial infarction and arrhythmia, indicating poor prognosis. Research suggests that HRV also plays an important role in patients with chronic pain disorders such as fibromyalgia, chronic neck and shoulder pain, trigeminal neuralgia, post-herpetic neuralgia, and others [9].

Pain is the main clinical manifestation of CNNP patients, and chronic pain can in turn induce anxiety. It has been reported that acute/chronic pain and anxiety are important variables causing changes in HVR [10]. These changes reduce HRV via sympathetic activation and parasympathetic suppression. The atlantoaxial (C1–C2) complex plays a pivotal role in cervical biomechanics. The atlantoaxial deviation has been implicated in CNNP patients due to its potential to compromise neurovascular structures or induce compensatory postural adaptations. Previous literature suggests that this deviation can affect HRV by affecting autonomic dysfunction [11]. However, there is no direct measurement of atlantoaxial deviation. We innovatively introduced a measure of atlantoaxial deviation and correlated it with HRV, which has not previously been addressed in the literature.

The aims of this study were a) to assess the differences in HRV between the healthy population and patients with CNNP; b) to explore the indicators associated with HRV in CNNP; and c) to determine whether HRV can objectively indicate the exacerbation of pain and identify patients with CNNP accompanied by anxiety. This study may help to clinically evaluate CNNP comprehensively and objectively, and provide a basis for refined individualized treatment for patients with CNNP.

## Materials and methods

The study was reviewed and approved by the Ethics Committee of the first affiliated hospital of Zhejiang Chinese Medical University (ethics number:2021-K-491–01 19/07/2021) and registered in the International Traditional Medicine Clinical Trial Registry (registration number: ITMCTR2024000187). All participants provided written informed consent before being included in the study. Study volunteers (healthy controls and patients with chronic cervical pain) were enrolled between January 2023 and December 2023. All data were collected at the Department of Tuina and Health Management Center, the first Affiliated Hospital of Zhejiang University of Traditional Chinese Medicine. This study followed the CONSORT (Consolidated Standards of Reporting Trials) checklist guidelines. All methods adhered to the tenets of the Declaration of Helsinki and relevant guidelines and regulations.

### Participants

#### Inclusion criteria and recruitment.

**Inclusion Criteria of CNNP group:** ①Adults aged 20–50 years; ②Chronic nonspecific neck pain (CNNP) persisting >12 weeks; ③Pain intensity ≥2/10 on the Visual Analog Scale (VAS).

**Recruitment:** Patients were recruited from inpatient ward of Tuina department, the first Affiliated Hospital of Zhejiang University of Traditional Chinese Medicine.

**Inclusion Criteria of control group:** ①Adults aged 20–50 years; ②No neck pain or cervical spine-related symptoms for ≥12 weeks prior to enrollment.

**Recruitment:** Controls were selected from the Health Management Centre of the First Affiliated Hospital of Zhejiang Chinese Medical University, representing a general population undergoing routine health screenings.

#### Exclusion criteria.

Participants were excluded if they met any of the following: ①Medical conditions affecting ANS/HRV: a) severe arrhythmia, coronary artery disease, or pacemaker implantation; b) Neurological disorders (e.g., Parkinson’s disease, diabetic neuropathy); c) Acute infections or systemic inflammatory diseases; ②Medications influencing ANS function: We classified medication that affect autonomic function into two categories: direct and potential effects. Beta-blockers, calcium channel blockers, anticholinergics, sedatives or antidepressants have a direct confounding effect on autonomic function, and those taking any of the above medications will usually be excluded. NSAIDs, muscle relaxants, analgesics or anti-inflammatory medications have a potential effect on autonomic function, and those taking any of the above medications within 48 hours of HRV testing are also excluded. ③HRV data quality: 20% of RR intervals disturbed in repeated HRV measurements (e.g., artifacts, ectopic beats); ④Educational level may correlate with health literacy and pain management behaviours, which could indirectly influence self-reported pain scores. Therefore, illiterate patients are usually excluded.

### Experimental procedure

**3 days prior to the experiment:** Participants received detailed instructions via WeChat (or equivalent messaging platforms) outlining: a) Dietary restrictions: Avoid tea, coffee, alcohol, and smoking for 12 hours before the test. b) Medication discontinuation: Stop ANS-affecting drugs (e.g., beta-blockers, anticholinergics) 24–48 hours before testing. c)Activity guidelines: Refrain from strenuous exercise and emotional stressors on the test day.

**The experimental day: *Participant Assessment:*** Participants completed the Case Report Form (CRF) in a separate room prior to the test. The CRF was pre-designed by the Centre for Clinical Evaluation of the first affiliated hospital of Zhejiang Chinese Medical University and consisted of four sections: demographics, pain characteristics, comorbidities and psychological assessment, with the healthy control group completing only the demographics section and the CNNP group completing all sections. When completed, the information was placed in a sealed envelope and given to a statistician for further analysis.

**Cervical Spine Imaging:** After collecting baseline data, we performed cervical spine imaging for the CNNP group. Healthy controls did not undergo imaging. **a) Cervical X-ray:** Technicians acquire digital radiography (DR) images using a study-specific digital radiography system (YSIO X. pree, manufactured by Siemens AG, Germany). Radiologists use a picture archiving and communication system (GE-PACS/RIS) to measure various parameters. **b) Cervical MRI:** Magnetic resonance images are taken by specialist technicians using a 3T whole-body scanner. The scanner is specifically designed for cervical disc herniation studies. Radiologist determine the presence of a lumbar disc herniation using GE-PACS/RIS.

**Heart Rate Variability (HRV) Measurement*:* a)Pre-Test Preparation: 12-hour restrictions:** No caffeine, alcohol, tea, smoking, or stimulants.

**24–48-hour restrictions:** Discontinued ANS-affecting medications (e.g., beta-blockers, anticholinergics).

**pre-test 5-minute**: Practice session to familiarize participants with spontaneous breathing and minimize anxiety.

**b) Immediate preparation:** Participants rested in a supine position for 10 minutes in a quiet, climate-controlled room (24–26°C, 40–50% humidity).

**c) HRV Recording:** Equipment: HRV was determined using the EDAN 12-channel ambulatory ECG acquisition box and the corresponding ambulatory ECG analysis system (Model: SE-2012, Sampling Frequency: 128 Hz, A/D Conversion Accuracy: 16 bits, Manufacturer: Shenzhen Ribbon Precision Instrument Co). **Participant:** HRV test needs to be assessed in both the CNNP group and the health control group. **Timing:** The gold-standard for measuring HRV is in the midnight, while the participant is asleep, since this is the time of maximum vagalization [12]. This timing is not available with our equipment, so our recordings were collected in the morning on awakening, when the participant should be in the vagally predominant, and participants were requested to measure their HRV for five minutes in advance to ensure their breathing spontaneously [13]. **Manipulation:** The three examiners divide the work into turns, one for the patient’s twelve-lead placement, the other is responsible for time recording, press the start button when the timer to record the dynamic electrocardiogram, the detection time is 5 min. the other personnel will record the electrocardiogram signal through the A/D conversion and input into the computer by the computer software to automatically read the data and generate the HRV time-domain analysis and frequency-domain analysis of information. **Duration:** A 5-minute acclimatization period preceded the formal 5-minute HRV recording to stabilize ANS activity. **Quality Control:** Excluded participants if >20% of RR intervals were artifacts/ectopic beats. Tests were repeated if poor signal quality was detected. To minimise the autonomic effect of body position on HRV, all HRV measurements were taken in the supine position [14].

### Data acquisition

**Baseline Data Collection: a) Demographics:** Age, gender, education, Body Mass Index and heart rate. **b) Pain Characteristics:** duration (>12 weeks), intensity (VAS score >2/10), pain localization and functional limitations. **c) Comorbidities:** documented conditions (e.g., hypertension, Parkinson’s disease) that may influence ANS function or pain perception. **d) Psychological Evaluation:** Self-Rating Anxiety Scale (SAS): A validated 20-item questionnaire quantifying anxiety symptoms. Each question can be scored from 1 to 4, with a score exceeding 50 indicating that the patient is anxious. **e) Medications taking:** Whether the patient has been taking medication that affects autonomic function, and if so, the dose, duration, and discontinuation will be recorded.

**Cervical Spine Imaging Data Collection: a) Atlantoaxial instability:** the distance between the lateral mass of the atlas and the axis of the odontoid, the difference between the apex of the spinous process and the axis of the odontoid process. **b) Degenerative changes in the cervical spine:** the curvature of the cervical vertebrae, and the osteoproliferation. c) cervical disc herniation

**HRV Data Collection:** Time-domain methods and frequency-domain methods represent the most common methods of HRV data collection. **a) Time-domain methods:** the standard deviation of all normal-to-normal beat intervals (SDNN) in ms, indicating the variation within R-wave time intervals. b) **The frequency parameters:** low-frequency (LF: 0.04–0.15 Hz), high-frequency (HF: 0.15–0.4 Hz) and their ratio (LF/HF)

### Analysis of HRV and imageological examination

In general, the most commonly used parameters for HRV analysis include SDNN, LF, HF, and LF/HF. LF and HF were selected to assess the ANS response. Pagani [15] introduced LF, HF, and LF/HF as measures to evaluate the reciprocal activity of the sympathetic and parasympathetic nervous system. HF was believed to be related to the cardiac parasympathetic function and LF to the cardiac sympathetic activity. The relative power of HF and LF was expressed as LF/HF. This was used to assess the relative activity of the sympathetic and parasympathetic nerves. SDNN, on the other hand, was a time-domain indicator reflecting overall ANS activity (Table 1).

**Table 1:** HRV variables and their definitions, categorized by domain.

Time-Domain Variables		
Variable	Brief Definition	Mean
SDNN	Standard deviation of all normal RR intervals	overall HRV
Frequency-Domain Variables		
LF	Power in low-frequency range (0.04–0.15 Hz)	mixed autonomic influence (sympathetic + parasympathetic)
HF	Power in high-frequency range (0.15–0.4 Hz)	parasympathetic activity
LF/HF	Ratio of LF to HF power	sympathovagal balance

**Fig 1:**
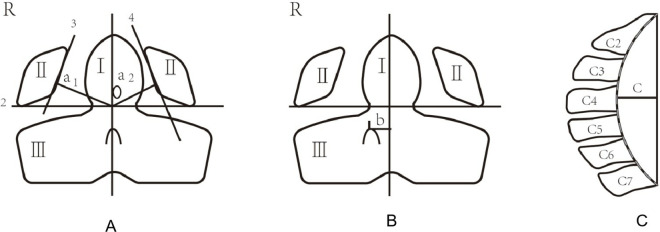
Imaging measurement methods of atlantoaxial deviation, axis rotation, and physiologic cervical curve. a1: vertical distance from odontoid process to right lateral mass of atlas, a2: Vertical distance from odontoid process to left lateral mass of atlas, b: The vertical distance from the spinous processes to the axis of odontoid, c:The horizontal transverse line at the widest point between the two lines.

This study analyzed the atlantoaxial deviation and odontoid rotation to effectively evaluate atlantoaxial instability. The ratio method a(a1/a2) was employed as an indicator of the degree of atlantoaxial deviation. In normal conditions, the atlantoaxial was centered without distortion (a1/a2 = 1). If the ratio was greater than 1 (a1/a2 > 1), the odontoid process was located on the left side, and if the ratio was less than 1 (a1/a2 < 1), the odontoid process was located on the right side (Fig 1A). In addition, the non-collinear level of the odontoid and spinous processes was used to evaluate the rotation degree of the axis. The collinearity of the odontoid and spinous processes indicated the absence of odontoid rotation (b = 0). In contrast, non-collinear odontoid and spinous processes indicated rotation to the left or right (b > 0 or b < 0, respectively) (Fig 1A).

This study analyzed the curvature of the cervical vertebrae and the osteoproliferation to reflect the degree of degeneration of the cervical spine. Borden’s measurement was used to evaluate cervical curvature [16]. A straight line was drawn from the posterior superior edge of the second cervical vertebra to the posterior lower edge of the C7 vertebral body. Then, a line was drawn along the posterior edge of each vertebra. The horizontal transverse line at the widest point between the two lines represents the depth of the physiological cervical curve (c) (Fig 1B). The typical range for value (c) was 12 ± 5 mm. In this study, c > 17mm indicated an excessive curvature. Conversely, c < 7mm indicated a reduced curvature(Fig 1C). Osteoproliferation and cervical disc herniation were evaluated by assigning values. The presence of osteoproliferation was assigned a value of 1, while the absence of osteoproliferation was assigned a value of 0.

This study used an assignment method to reflect cervical disc herniation. If the individual had a herniated cervical disc, a value of 1 was assigned, and conversely, a value of 0 was assigned.

### Sample size calculation

The effect size (d) of HRV in both the healthy and CNNP groups was 0.48, based on previous references [17] and pre-tests conducted by the project team. Jaykaran [18] reports the formula for estimating the sample size for a quantitative variable as follows:


$$\text{Sample size}=\frac{{{2(\text{Z}}_{\alpha /2}+{\text{Z}}_{\beta })}^{2}}{{\text{d}}^{2}}$$

Z_a/2_ = Z_0.05/2 _= Z_0.025_ = 1.96 (From Z table) with a type Ⅰ error of 5%

Z_β_=0.84

d = 0.48

Participants were assigned to the CNNP and healthy control groups on a 1:1 basis. According to the formula, the sample size was further increased by 20% to account for potential factors such as lost visits and attrition, with each group initially comprising 70 cases. Therefore, study subjects were required to have a minimum of 84 cases per group.

### Statistical analysis

The statistical analyses were performed using IBM SPSS Statistics software (version 29). Normality of data distribution was evaluated using the Shapiro-Wilk test. For variables adhering to a normal distribution, descriptive statistics are presented as mean (standard deviation, SD) accompanied by a confidence interval (CI). In contrast, non-normally distributed variables are summarized as median and interquartile range (IQR). Between-group comparisons were conducted using independent samples t-tests for normally distributed data, with effect sizes reported as Cohen’s d. For non-normal data, the Mann-Whitney U test was applied, and the effect size was quantified using the rank-biserial correlation (r). Relationships between heart rate variability (HRV) indices (time and frequency domains) and clinical/demographic variables (e.g., age, Visual Analogue Scale [VAS] scores, Self-Rating Anxiety Scale [SAS] scores) were assessed via Pearson’s correlation coefficient (r) for normally distributed data. Correlation strengths were interpreted as weak (|r| < 0.3), moderate (|r| = 0.3–0.5), or strong (|r| > 0.5). A two-tailed significance threshold of α ≤ 0.05 was applied for all inferential analyses to determine statistical significance.

## Results

### Characteristics of participants

**Fig 2:**
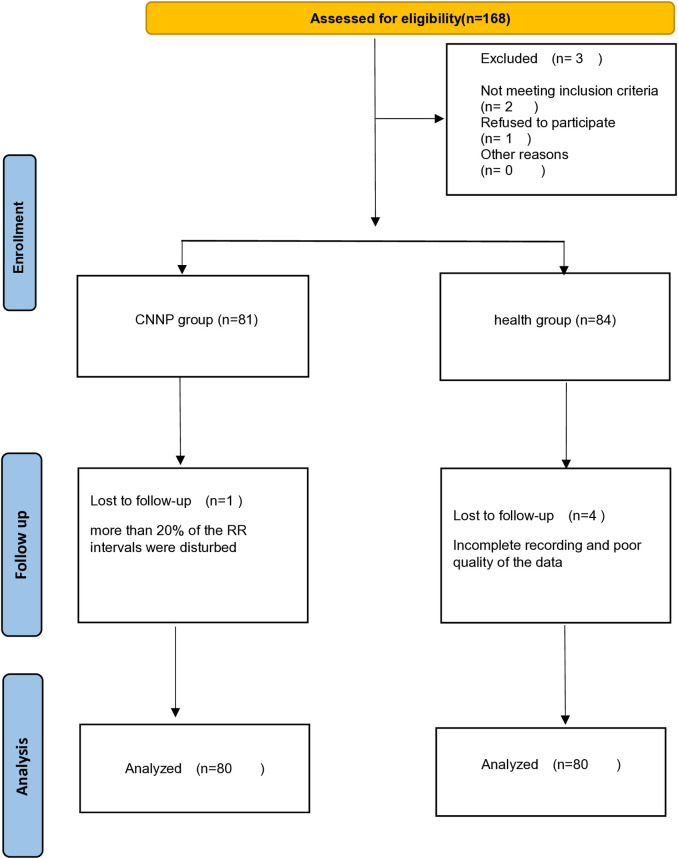
Flow diagram of study participants.

A total of 168 participants (84 patients with chronic nonspecific neck pain and 84 healthy controls) were recruited for this study. Three patients with cardiovascular diseases (two patients with a long-term beta-blocker course) were excluded from the study. The HRV test results of one patient showed that more than 20% of the RR intervals were disturbed, and were excluded. Four of the controls were excluded due to incomplete recording of the pulse or poor quality of the data. Finally, 160 participants (80 patients with chronic nonspecific neck pain and 80 healthy controls) were included in the analysis (Fig 2).

No statistically significant differences in age (independent samples t-test; t = −0.153, p = 0.13), gender (Chi-Square test; χ^2^ = 0.42, p = 0.52), education (independent samples t-test; t = 1.87, p = 0.06), BMI (independent samples t-test; t = −0.45, p = 0.66), and heart rate (independent samples t-test; t = −1.21, p = 0.21) were observed. A total of 52 patients with chronic neck pain were currently using painkillers and 69 had used painkillers, but all were asked to stop taking their medication within 48 hours of the start of the trial. At baseline, 13 patients were taking low-dose tetracyclic antidepressants and were asked to stop the medication 48 hours before the start of the trial in order not to affect the results. None of the healthy controls were taking any medication at the time of this study. The enrolled participants were taking medications that could not have affected the autonomic nervous system (Table 2).

**Table 2:** The demographics and characteristics of study paticipation.

Characteristics	Patients with CNNP(n = 80)	Health control (n = 80)	*P*-value
age(years), Mean(SD) [CI]	34.43(9.10)[32.49;36.54]	36.28(5.88)[34.95;37.58]	0.13
Gender (m/f) Education(years) (SD) [CI]	15/65 16.01(3.15)[15.27;16.68]	17/63 15.10(3.04)[14.46;15.75]	0.06
Body Mass Index(BMI) (kg/m^2^) (SD) [CI]	23.21(1.78)[22.80;23.62]	23.33(1.65)[22.99;23.72]	0.66
Heart rate^‌^(BMP) (SD) [CI]	72.10(6.32)[70.82;73.53]	73.28(5.93)[72.08;74.51]	0.21

SD:Standard deviation, CI:confidence interval, CNNP:Chronic nonspecific neck pain, m/f:male/female.

### The differences in HRV between two group

**Fig 3:**
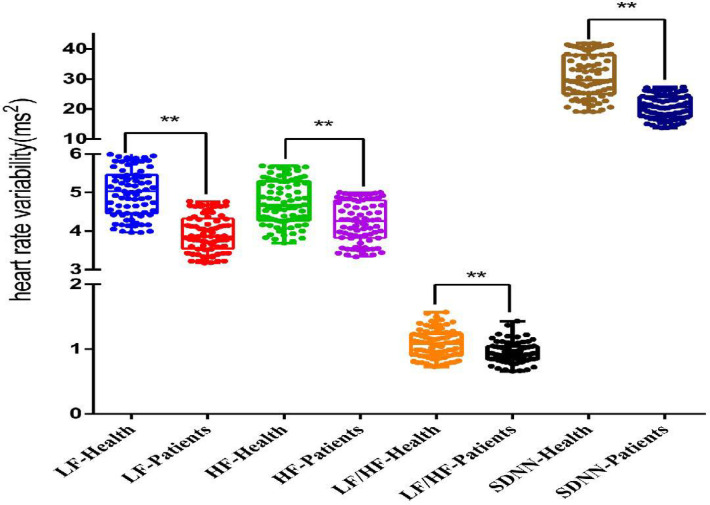
Heat Rate Variability between Health Control and Patients with Chronic Nonspecific Neck Pain. **: *P* < 0.00, LF: low frequency, HF: high frequency, SDNN: standard deviation of all normal-to-normal QRS intervals.

The heart rate variability is presented in (Fig 3), which depicts the SDNN, LF, HF, and LF/HF results for both the CNNP and healthy control groups. The SDNN, LF, HF, and LF/HF results of the two groups followed a normal distribution according to the normality test. The CNNP group exhibited a significantly lower (*P* < 0.00) SDNN by 10.24 (95%CI −12.03 to −8.45), LF by 1.07 (95%CI −1.24 to −0.90), HF by 0.49 (95%CI −0.67 to −0.32) and LF/HF by 0.14 (95%CI −0.19 to −0.08) compared to the healthy control group.

### The indicators associated with HRV in CNNP

Patients with CNNP showed a significant correlation between HRV and VAS, SAS, atlantoaxial deviation, axis rotation, and cervical disc herniation. However, no correlation was found between HRV and age, disease progression, osteoproliferation, and cervical spine curvature. Furthermore, no significant correlations were observed when atlantoaxial deviation was less than 0.01 mm and rotation of the axis was less than 2 mm (Table 3).

**Table 3:** Correlations between HRV indexes and clinical outcome in patients with CNNP.

HRV indexes	Variable	Pearson's r (95%CI)	*P-*Value
HF	Age	−0.19(−0.4 to 0.36)	0.09
	Atlantoaxial deviation	0.24(0.01 to 0.45)	0.03
	>0.1mm	0.42(0.17 to 0.64)	<0.01
	<0.1mm	−0.2(−0.5 to 0.13)	0.2
	Course of disease	0.01(−0.22 to 0.24)	0.92
	SAS	−0.24(−0.48 to −0.2)	0.03
	Osteoproliferation	−0.01(−0.25 to 0.25)	0.98
	VAS	−0.43(−0.66 to- 0.14)	<0.01
	Cervical curvature	0.1(−0.13 to 0.32)	0.42
	Axis rotation	−0.22(−0.42 to −0.03)	0.04
	>2mm	−0.32(−0.54 to −0.1)	0.03
	<2mm	−0.04(−0.35 to 0.23)	0.81
	Herniated disc	0.27(0.06 to 0.5)	0.02
LF	Age	−0.19(−0.4 to 0.04)	0.08
	Atlantoaxial deviation	0.23(−0.01 to 0.44)	0.04
	>0.1mm	0.43(0.19 to 0.62)	<0.01
	<0.1mm	−0.2(−0.5 to 0.1)	0.24
	Course of disease	0.26(−0.23 to 0.27)	0.82
	SAS	−0.29(−0.49 to −0.05)	0.02
	Osteoproliferation	−0.03(−0.2 to 0.26)	0.83
	VAS	−0.43(−0.68 to −0.18)	<0.01
	Cervical curvature	0.1(−0.15 to 0.32)	0.41
	Axis rotation	−0.25 (−0.44 to −0.06)	0.03
	>2mm	−0.35 (−0.57 to −0.14)	0.04
	<2mm	−0.11 (−0.39 to 0.14)	0.53
	Herniated disc	0.28 (0.03 to 0.5)	0.01
LF/HF	Age	−0.18(−0.39 to 0.46)	0.11
	Atlantoaxial deviation	0.24(−0.01 to 0.45)	0.04
	>0.1mm	0.43(0.13 to 0.69)	<0.01
	<0.1mm	−0.23(−0.45 to 0.05)	0.17
	Course of disease	0.46(−0.21 to 0.26)	0.69
	SAS	−0.26(−0.51 to −0.05)	0.02
	Osteoproliferation	−0.02(−0.24 to 0.22)	0.84
	VAS	−0.44(−0.68 to −0.18)	<0.01
	Cervical curvature	0.08(−0.16 to 0.29)	0.48
	Axis rotation	−0.22 (−0.41 to −0.03)	0.04
	>2mm	−0.04 (−0.58 to −0.17)	0.02
	<2mm	−0.01 (−0.35 to 0.26)	0.93
	Herniated disc	0.25 (0.16 to 0.47)	0.03
SDNN	Age	−0.19(−0.41 to 0.03)	0.09
	Atlantoaxial deviation	0.23(−0.01 to 0.45)	0.03
	>0.1mm	0.42(0.17 to 0.63)	<0.01
	<0.1mm	−0.17(−0.5 to 0.15)	0.31
	Course of disease	0.14(−0.24 to 0.25)	0.9
	SAS	−0.24(−0.47 to −0.01)	0.03
	Osteoproliferation	−0.01(−0.24 to 0.23)	0.96
	VAS	−0.43(−0.68 to −0.18)	<0.01
	Cervical curvature	0.1(−0.16 to 0.32)	0.42
	Axis rotation	−0.22 (−0.41 to −0.03)	0.04
	>2mm	−0.3 (−0.6 to −0.04)	0.04
	<2mm	−0.05 (−0.36 to 0.22)	0.77
	Herniated disc	0.26 (0.01 to 0.5)	0.02

### HRV analysis of different levels of pain and combined anxiety status in CNNP patients

Patients with CNNP were divided based on the VAS scores, including the mild pain group (VAS < 3) and the severe pain group (VAS ≧ 3). After the normality test, the VAS scores of the two groups followed a normal distribution. The mild pain group (VAS < 3) showed a significantly higher (p < 0.00) SDNN by 2.31 (95%CI 0.71 to 3.9), LF by 2.31 (95%CI 0.71 to 3.9) and HF by 0.65 (95%CI 0.37 to 0.82) than severe pain group (VAS ≧ 3) (Table 4).

**Table 4:** Comparison of HRV between mild pain group (VAS < 3) and moderate to severe pain group (VAS ≥ 3).

	VAS < 3 (n = 36)	VAS ≥ 3 (n = 44)	Cohen’s d	*P*-value
LF (SD) [CI] in ms^2^	5.34(0.56)[5.15;5.52]	4.74(0.52)[4.60;4.89]	1.12	0.01
HF (SD) [CI] in ms^2^	5.06(0.51)[4.87;5.22]	4.46(0.48)[4.34;4.60]	1.22	0.01
LF/HF (SD) [CI]	1.06(0.01)[1.05;1.06]	1.06(0.01)[1.06;1.07]	/	0.75
SDNN (SD) [CI] in ms	20.16(4.17)[18.79;21.58]	17.84(2.57)[17.12;18.64]	0.69	0.01

LF: low frequency, HF: high frequency, SDNN: standard deviation of all normal-to-normal beat intervals, CI: confidence interval, VAS: Visual Analogue Scale, HRV: heart rate variability.

Based on the SAS score, patients with CNNP were divided into those without anxiety (SAS < 50) and those with anxiety (SAS ≧ 50). After testing for normality, the SAS scores of the two groups followed a normal distribution. The group without anxiety showed a significantly higher (p < 0.01) SDNN by 2.19 (95%CI 0.751 to 3.86), LF by 0.35 (95%CI 0.08 to 0.61), and HF by 0.32 (95%CI 0.77 to 0.57) than the group with anxiety (Table 5).

**Table 5:** Comparison of heart rate variability between patients with anxiety and non-anxiety.

	Anxiety group (n = 39)	Non-anxiety group (n = 41)	Cohen’s d	*P*-value
LF (SD) [CI] in ms^2^	5.19(0.57)[4.99;5.35]	4.84(0.61)[4.66;5.01]	0.59	0.01
HF (SD) [CI] in ms^2^	4.89(0.54)[4.71;5.05]	4.57(0.56)[4.41;4.73]	0.58	0.01
LF/HF (SD) [CI]	1.06(0.01)[1.05;1.06]	1.05(0.01)[1.05;1.06]	/	0.75
SDNN (SD) [CI] in ms	21.82(3.67)[20.59;22.86]	19.63(3.85)[18.52;20.69]	0.58	0.01

LF: low frequency, HF: high frequency, SDNN: standard deviation of all normal-to-normal beat intervals, CI: confidence interval, SD: standard deviation.

## Discussion

In our study, CNNP patients exhibited a predominance of vagal inhibition, characterized by reduced activity of the parasympathetic nervous system (PNS). This was evidenced by lower SDNN, LF, HF, and other indices of HRV (established markers of vagal activity), suggesting that PNS regulation was reduced in patients with CNNP. Previous studies had also shown lower HRV metrics may exacerbate pain perception and anxiety [19], which was confirmed in this study. However, the relationship between atlantoaxial instability and HRV index was not investigated. Finally, we found HRV may be an objective indicator of pain exacerbation, anxiety in CNNP patients in our study.

### Differences in HRV between two groups may indicate ANS dysfunction in CNNP patients

A few studies have reported that autonomic nervous system disorders are more common in patients with CNNP than cervical pain and radiculopathy due to nerve root or spinal cord compression [5,20]. On one hand, Tracey [21] observed the vagal inhibition, reflected by reduced RMSSD and HF power, aligns with the vagus nerve’s role in the cholinergic anti-inflammatory pathway. Our study also found that SDNN, LF, HF, and LF/HF were significantly lower in patients with CNNP compared with the healthy population, suggesting a decrease in overall PNS function in patients with CNNP, with a predominance of vagal inhibition. Pavlov [22] found that diminished vagal tone may exacerbate pro-inflammatory cytokine release (e.g., IL-6, TNF-α), perpetuating nociceptive sensitization in chronic neck pain. This mechanistic insight underscores HRV’s potential not only as a biomarker but also as a therapeutic target, where interventions like transcutaneous vagus nerve stimulation (tVNS) could be explored for pain modulation. On the other hand, Sustained vagal inhibition may have more profound effects beyond pain. Studies, including epidemiologic cohort studies and case-controlled studies, have already shown correlations between vagal inhibition and the progression of atherosclerosis [23], plaque formation [24], and even cardiovascular (CV) events [25]. One study also revealed a negative correlation between SDNN, LF in HRV, and resting diastolic blood pressure [26]. In this study, CNNP patients with cardiovascular comorbidities were excluded and still showed a significant decrease in PNS function. PNS dysfunction leading to cardiovascular disease was well established before [27]. It was hypothesised that CNNP results in an imbalance in the regulatory function of PNS, which in turn indirectly contributes to the development of cardiovascular disease. A study confirmed our conclusion that the incidence of acute coronary syndrome (ACS) was 1.13 times higher in CNNP patients than in non-CNNP patients and that treatment of CNNP patients reduced the incidence of ACS [28].

### Atlantoaxial instability was found to be correlated to HRV

Previous studies have shown that a range of indicators such as pain and anxiety in CNNP patients correlate with HRV. This study innovatively elucidates the correlation between atlantoaxial instability and HRV, uncovering the relationship between the two variables. Compared with other cervical vertebrae, the upper cervical segment, centred on the atlantoaxial joint, is less stable because it lacks stabilising structures such as intervertebral discs, has no posterior synovial joints and is held in place only by ligaments [29]. However, the atlantoaxial joint is the most flexible functional unit of the spine, coordinating approximately 50% of the rotational functions of the cervical spine. This makes the atlantoaxial spine highly susceptible to instability. Atlantoaxial instability typically manifested as atlantoaxial deviation and odontoid rotation. Studies have shown that patients with upper cervical instability have positive sympathetic symptoms such as dizziness, headaches and panic attacks [30] and cervical angina was first described in 1934 by Nachla [31] and was caused by atlantoaxial instability, suggesting the involvement of sympathetic function [32]. The upper cervical ganglion (SCG) is anatomically close to the upper cervical segment and is the largest ganglion in the cervical sympathetic trunk with the largest number of divisions, and the divisions of the SCG are connected to the lower vagus ganglion, etc. When the upper cervical segment becomes unstable, the SCG stimulates the lower vagus ganglion, leading to autonomic dysfunction [33]. Farrell et al [34] found that after upper cervical mobilization, the vagal “rebound effect” occurred, with an initial increase in rMSSD, the most important temporal measure of vagal tone in the HVR, and an eventual decline. Budgell et al [35] also found that the ratio of the LF/HF parts of the power spectrum of heart rate variability increased transiently, which may reflect a shift in the balance between sympathetic and parasympathetic output to the heart. In our study, mild upper cervical instability was not correlated with the HRV index. However, when the instability reached a certain level (atlantoaxial deviation > 0.1 mm or rotation of the axis > 2 mm), a significant correlation with the HRV index was found. Presumably, atlantoaxial instability stimulates the SCG, causing a dysfunction of the ANS, resulting in changes in cardiac output.

### HRV indicates pain levels and identifies the presence of anxiety in patients with CNNP

Self-report measures are currently the most commonly used method for pain [36]. For instance, pain intensity can be measured by applying the visual analog scale (VAS) and numerical rating scale (NRS) [37]. However, this method lacks objectivity. With the deepening research on the relationship between pain and ANS, a growing number of studies have employed HRV as a physiological index of the ability of the organism to respond flexibly to stress, such as pain. Burton et al.[38] used the LF/HF ratio as one of the indices to evaluate visceral and cutaneous pain. Evans, D.R. et al [39] used HF, LF, and the LF/HF ratio as indexes to assess pain tolerance. Appelhans and Luecken [40] found that HRV-LF may reflect sympathetic activity and is negatively correlated with pain sensitivity. The results of the present study revealed that the HRV indicators HF, LF, HF/LF, and SDNN were correlated with VAS in CNNP patients. In addition, the LF, HF, and SDNN were significantly lower in CNNP patients in the moderate to severe pain group compared to the mild pain group. There are extensive interactions between neural structures involved in nociception and autonomic control [41], and the vagus nerve plays an important role in both the upward pathway of transmission of noxious stimuli to the center and the downward inhibitory pathway within the dorsal horn of the spinal cord; a decrease in vagal activity may result in a larger input of noxious stimuli through the thalamic tract of the spinal cord [42]. HRV was significantly correlated with the level of pain in CNNP patients and can somewhat objectively reflect the pain of CNNP patients.

Anxiety is the most prevalent psychiatric disorder. Studies have shown that patients with anxiety disorders have a 3–5 times higher relation of cardiovascular disease compared to normal subjects [43]. A growing number of studies have reported that patients with anxiety disorders have reduced HRV compared to normal subjects [44], but the effect of anxiety on ANS disorders in CNNP patients remains poorly understood. The results of the present study showed a correlation between HRV and ANS dysfunction in CNNP patients, revealing significantly lower HF, LF, and SDNN in CNNP patients with comorbid anxiety compared to the non-anxious group. These findings suggest that the presence of anxiety may further exacerbate ANS dysfunction in CNNP patients, with predominating vagal inhibition. This finding supports the neurovisceral integration model proposed by Thayer et al.[45], which suggests that reduced vagal tone may be the ultimate pathway linking negative affective states to poor physical health. Therefore, HRV may help us to identify CNNP patients with anxiety at an early stage, and the psychological adjustment of patients may also play an important role in improving the overall functioning of the ANS and the prognosis of patients.

## Conclusions

Our findings position HRV as a viable, non-invasive tool for identifying autonomic dysfunction in CNNP patients. Clinically, HRV monitoring could guide personalized interventions—such as individualised assessment of treatment efficacy and early identification of CNNP patients combined with anxiety. Future research should integrate HRV with multimodal assessments (e.g., cervical imaging, inflammatory biomarkers) to unravel the bidirectional pathways linking ANS dysregulation, biomechanical stress, and chronic pain.

## Supporting information

S1 Table The demographics and characteristics of study paticipation. (XLSX)

S2 Table Correlations between HRV indexes and clinical outcome in patients with CNNP. (XLSX)

S3 Table Comparison of HRV between mild pain group (VAS < 3) and moderate to severe pain group (VAS ≥ 3). (XLSX)

S4 Table Comparison of heart rate variability between patients with anxiety and non-anxiety. (XLSX)

S1 File All the data of this study. Group1 = Health Control (They only measured basic information and indicators of HRV; “/” means the variable was not measured). Group2 = Patients with Chronic Nonspecific Neck Pain.
(XLSX)
